# Efficacy of Urine Dipstick Test in Diagnosing Urinary Tract Infection and Detection of the blaCTX-M Gene among ESBL-Producing *Escherichia coli*

**DOI:** 10.3390/diseases9030059

**Published:** 2021-08-27

**Authors:** Rubina Gurung, Sanjib Adhikari, Nabaraj Adhikari, Sanjeep Sapkota, Jid Chani Rana, Binod Dhungel, Upendra Thapa Shrestha, Megha Raj Banjara, Prakash Ghimire, Komal Raj Rijal

**Affiliations:** 1 Central Department of Microbiology, Tribhuvan University, Kirtipur 44600, Kathmandu, Nepal; gurungrubina16@gmail.com (R.G.); sanadh26@gmail.com (S.A.); adhikarinaba13@gmail.com (N.A.); bwith.binod@gmail.com (B.D.); upendrats@gmail.com (U.T.S.); banjaramr@gmail.com (M.R.B.); prakashghimire@gmail.com (P.G.); 2 Department of Microbiology, Birendra Multiple Campus, Tribhuvan University, Bharatpur 44200, Chitwan, Nepal; sansanjeep123@gmail.com; 3 Department of Microbiology, Bharatpur Hospital, Bharatpur 44200, Chitwan, Nepal; sheepath@yahoo.com

**Keywords:** *Escherichia coli*, ESBL, sensitivity, specificity, PPV, NPV, *bla*_CTX-M_

## Abstract

A urine dipstick test used for prompt diagnosis of urinary tract infection (UTI) is a rapid and cost-effective method. The main objective of this study was to compare the efficacy of the urine dipstick test with culture methods in screening for UTIs along with the detection of the *bla*_CTX-M_ gene in extended spectrum β-lactamase (ESBL)-producing *Escherichia coli*. A total of 217 mid-stream urine samples were collected from UTI-suspected patients attending Bharatpur Hospital, Chitwan, and tested by dipstick test strip (COMBI-10SL, Germany) prior to the culture. *E. coli* isolates were identified by standard microbiological procedures and subjected to antimicrobial susceptibility testing by Kirby Bauer disc diffusion method following CLSI guideline. Primary screening of ESBL-producing *E. coli* isolates was conducted using ceftriaxone, cefotaxime and ceftazidime discs and phenotypically confirmed by combined disk diffusion test. Plasmid DNA of ESBL-producing strains was extracted by phenol-chloroform method and subjected to PCR for detection of the *bla*_CTX-M_ gene. Out of 217 urine samples, 48 (22.12%) showed significant bacteriuria. Among 46 (21.20%) Gram negative bacteria recovered, the predominant one was *E. coli* 37 (77.08%) of which 33 (89.19%) were multidrug resistant (MDR). *E. coli* isolates showed a higher degree of resistance towards cefazolin (62.16%) while 81.08% of the isolates were sensitive towards amikacin followed by nitrofurantoin (70.27%). Among 14 (37.84%) phenotypically confirmed ESBL isolates, only eight (21.62%) isolates carried the *bla*_CTX-M_ gene. Sensitivity, specificity, positive predictive value (PPV) and negative predictive value (NPV) of urine dipstick test were 43.75%, 77.51%, 35.59% and 82.91%, respectively. Besides, the use of dipstick test strip for screening UTI was associated with many false positive and negative results as compared to the gold standard culture method. Hence, dipstick nitrite test alone should not be used as sole method for screening UTIs.

## 1. Introduction

Urinary tract infection is the second most frequent infectious disease that probably affects one-half of all people during their lifetimes. UTIs simply mean the presence of microbial pathogens causing the infection within the urinary tract [1]. Each year, approximately 150 million people are diagnosed with UTI, costing the global economy of billions of US dollars [2]. It has been estimated that at least 40% to 50% of women will experience a minimum of one symptomatic UTI with roughly 27% to 48% of affected women experiencing recurrent UTIs [3]. A UTI is a severe public health problem caused by a variety of pathogens, but most frequently by *Escherichia coli* followed by *Klebsiella pneumoniae*, *Proteus mirabilis*, *Enterococcus faecalis*, *Staphylococcus saprophyticus*, *Staphylococcus aureus*, and *Pseudomonas aeruginosa* [4,5,6].

Urine culture is used as the reference standard to detect UTI; however, the culture method is expensive and time-consuming [7]. Urinalysis is one of the rapid screening techniques that have been used to diagnose UTIs, proteinuria, haematuria, and some other conditions [8]. Several tests are available for the diagnosis of UTIs, but the semi-quantitative culture of urine specimen is the only method for the detailed documentation of a bacterial urine infection [9]. The dipstick strip is considered to be one of the qualitative diagnostic tools [10] which include reagent pads such as nitrite, leukocyte esterase, specific gravity, pH, protein, glucose, ketones, bilirubin, urobilinogen and blood [11]. It is nevertheless associated with many false positive and negative results as compared to the gold standard culture method.

Beta-lactam drugs are among the most prescribed antibiotics that are used to treat diseases caused by MDR Enterobacteriaceae [12]. The excessive use of beta-lactam antibiotics has increased the expansion of resistant Enterobacteriaceae. Usually, ESBLs confer resistance to all penicillin, cephalosporins (except for cephamycins), and monobactams, being inhibited by β-lactam inhibitors [13,14] and they are the predominant sources of enterobacterial resistance to third and fourth generation cephalosporins. Among ESBLs observed in *E. coli,* the most commonly identified are enzymes from the family CTX-M (mostly CTX-M-1 lineage), followed by SHVs, and TEMs [15,16]. Plasmids have been found to confer drug resistance in their host bacteria by various mating processes such as conjugation, transduction and transformation with plasmid size [17]. They are responsible for the spread of antibiotic resistant genes in bacterial populations and the release of antibiotics into the environment will boost up R-plasmids, integrons and multidrug resistance [18].

In a developing country such as Nepal, specialised laboratories with diagnostic tools and techniques are limited and hence rapid tests such as urine dipstick tests are employed for the patients suspected of having UTIs. However, very few studies have reported the accuracy of these tests. Moreover, there is increasing evidence of drug resistant isolates in hospitals/clinical settings in Nepal [19,20,21,22]. The data available in the country necessary for marking the resistant patterns are not sufficient to describe the trend of antibiotic resistance. Therefore, the current study was designed to evaluate the efficacy of the urine dipstick test and for the detection of *bla*_CTX-M_ gene in Extended Spectrum β-lactamase (ESBL)-producing *E. coli* isolates.

## 2. Materials and Methods

### 2.1. Study Design and Setting

This was a hospital-based cross-sectional study conducted at Bharatpur hospital, Chitwan, Nepal, from January to April 2019. Bharatpur hospital is a government-funded tertiary care hospital situated in Bagmati Province in the mid-southern part of the country. The hospital has 600 beds and has been providing health services to around a 35 hundred-thousand population of eight major surrounding districts besides Chitwan itself.

### 2.2. Sample Collection, Processing and Identification

A total of 217 midstream urine samples were collected from UTI suspected patients of all ages and sexes visiting the hospital and processed in the Microbiology Laboratory of Bharatpur Hospital, Chitwan. Patients were asked to collect 10–20 mL of clean first-voided morning mid-stream urine in a sterile, dry, wide-necked, leakproof container and, once brought to the laboratory, the urine was tested first by using a dipstick test strip (COMBI-10SL, Analyticon Biotechnologies, Germany). Nitrite was considered as positive if there was a change in the colour of the dipstick from colourless to pink within 60 s. Urine culture was performed by using the strips calibrated to deliver 0.02 µL of urine on MacConkey and blood agar plates and incubated at 37 °C for 24 to 48 h. Following enumeration of the colonies, it was reported that there was significant bacteriuria if the growth was ≥10^5^ CFU/mL. The identification of bacterial isolates was conducted by conventional microbiological techniques that involved studying the colonial morphology, Gram staining and various biochemical tests [23]. The culture method was considered as gold standard to evaluate the performance of dipstick test.

### 2.3. Antimicrobial Susceptibility Testing

Antibiotic susceptibility tests of *E. coli* isolates were performed using a modified Kirby–Bauer disc diffusion method on Mueller Hinton Agar (MHA), as recommended by CLSI guidelines using an array of commonly prescribed antibiotics such as amikacin (30 µg), amoxiclave (30 µg), cefazolin (30 µg), cefixime (5 µg), ciprofloxacin (5 µg), ceftazidime (30 µg), ceftriaxone (30 µg), cefotaxime (30 µg), co-trimoxazole (25 µg), gentamicin (10 µg), nitrofurantoin (30 µg), nalidixic acid (30 µg), and piperacillin/tazobactam (30/6 µg) (CLSI 2018). The bacterial isolates showing resistance to three or more than three classes of antibiotics on MHA were reported as MDR isolates [24]. All the media and antibiotic discs used in the test were procured from HiMedia Laboratories, India.

### 2.4. Phenotypic Confirmation of ESBL Producers

The *E. coli* isolates were screened for possible ESBL production using ceftriaxone (30 µg), cefotaxime (30 µg), and ceftazidime (30 µg). The isolates showing a zone of inhibition (ZOI) of ≤25 mm for ceftriaxone, ≤27 mm for cefotaxime and ≤22 mm for ceftazidime were presumed to be the probable ESBL-producing strains (CLSI 2018). The suspected ESBL producers were subjected to a combined disk test comprised of cefotaxime (30 µg) and cefotaxime (30 µg) plus clavulanic acid (CA) (10 µg), ceftazidime (30 µg) and ceftazidime (30 µg) plus clavulanic acid (10 µg) for confirmation of ESBL production. An increase in ZOI of 5 mm for either antimicrobial agent tested in combination with CA versus its zone when tested alone confirmed ESBL production (CLSI 2018).

### 2.5. DNA Extraction and Amplification of bla_CTX-M_ Gene by PCR

An auxenic culture of *E. coli* grown on MacConkey agar was inoculated into 5 mL of Luria–Bertanii broth (Hi-media, India) and incubated at 37 °C for 24 h. Following incubation, the plasmid DNA was extracted by using the alkaline-lysis method [25]. The extracted DNA samples were suspended in 50 µL of TE buffer and stored at −20 °C and further used as the template in the PCR reaction. The primers used to detect the *bla_CTX-M_* specific gene have been enlisted (Table 1).

PCR amplification reactions were carried out in 50 Mm KCl, 10 Mm Tris-HCL (pH 9), 0.1% Triton X-100, 2 Mm MgCl_2_, a 200 µM concentration of each deoxynucleoside triphosphate, a 0.5 µM concentration of each primer, one bead of TaqBead hot start polymerase, and 5 µL of template DNA under thermal and cycling conditions for the *bla_CTX-M_* gene: initial denaturation at 94 °C for 2 min followed by 35 cycles of denaturation at 95 °C for 20 s, annealing at 51 °C for 30 s and extension at 72 °C for 30 s. After PCR amplification, 2.5 µL from each reaction was separated by electrophoresis in 1.5% agarose gel for 30 min at 100 V in 0.5× TBE buffer. DNA was stained with ethidium bromide (1 µg/mL) and the bands were detected using a UV-transilluminator [27]. The amplicon size for *bla_CTX-M_* was 544 bp [22].

### 2.6. Quality Control

Laboratory equipment such as an incubator, autoclave, hot air oven and refrigerator were regularly monitored for their efficiency. Reagents, media and antibiotic discs were meticulously checked for the expiry date and proper storage conditions. Media and reagents were properly labelled with the preparation date. Sterility and performance testing were carried out using standard control strains. For the standardization of the Kirby–Bauer test and performance testing of antibiotics and MHA, control strains of *E. coli* (ATCC25922) were tested primarily. Quality of sensitivity tests was maintained by maintaining the thickness of Mueller Hinton agar at 4 mm and the pH 7.2–7.4.

### 2.7. Data Analysis

Data were analysed by using SPSS software for windows (version 16). A value of *p* ≤ 0.05 was considered significant wherever applicable.

## 3. Results

### 3.1. Distribution of Isolates

**Figure 1:**
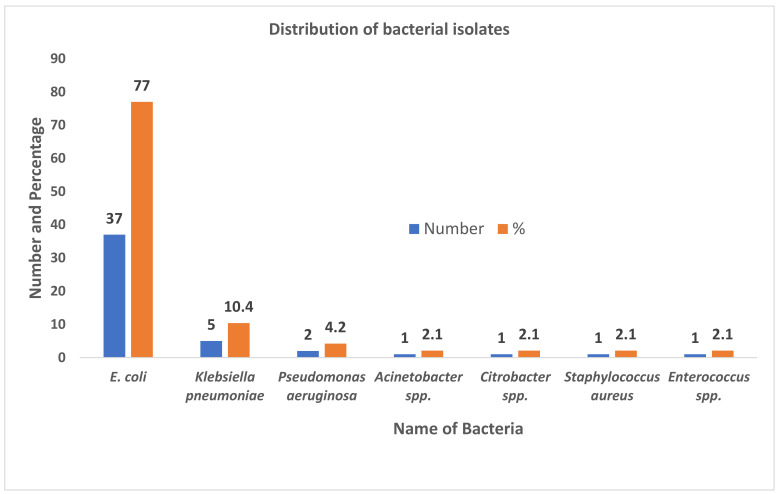
Distribution of bacterial isolates among UTI suspected patients attending Bharatpur hospital, Chitwan.

A total of 217 midstream urine samples were processed for bacterial investigations from where 48 isolates were recovered. *E. coli* was the most predominant bacteria 37 (77.08%) followed by *Klebsiella pneumoniae* 5 (10.42%) and *Pseudomonas aeruginosa* 2 (4.17%) (Figure 1).

### 3.2. Sensitivity, Specificity, Positive Predictive Value and Negative Predictive Value for Urinary Dipstick Test

The performance characteristics of the urinary dipstick test are shown (Table 2). Out of 217 urine samples, 48 samples showed significant growth of which 21 samples showed a nitrite test positive while 27 showed a nitrite test negative. Among total of 59 nitrite test positive samples, 38 growth negative samples showed nitrite test positives whilst 131 growth negative samples showed nitrite test negatives.

### 3.3. Association of UTI with Various Attributes

Of the 217 urine samples collected, 156 (71.89%) samples were from female whereas 61 (28.11%) samples were from male patients. Among 156 female samples, 39 (25.00%) showed significant growth, whereas of 61 samples from males, 9 (14.75%) showed significant growth (*p* > 0.05). Although a higher number of samples (103, 47.47%) were from the age group 20–39, prevalence of UTIs was also recorded from the age group 60–79 (*p* < 0.05). A large number of samples (138, 63.59%) were received from the out-patients who showed a higher incidence of UTIs (26.81%) compared to the in-patients (13.92%) (*p* < 0.05) (Table 3).

### 3.4. Antibiotic Susceptibility Pattern of Escherichia coli

The antibiotic susceptibility test (AST) of *E. coli* revealed that a majority of the isolates (30, 81.08%) were sensitive to amikacin followed by nitrofurantoin (26, 70.27%) and gentamicin (23, 62.16%). The most ineffective antibiotic was cefazolin resisted by 23 (62.16%) isolates. Out of 37 *E. coli* isolates subjected to AST, 33 (89.19%) isolates were MDR (Table 4).

### 3.5. Prevalence of ESBL Positive E. coli and bla_CTX-M_ Gene

A total of 37 *E. coli* isolates were screened for the ESBL production activity, of which 19 (51.35) isolates gave primary screening test positives. Fourteen isolates (37.84%) were further confirmed as ESBL producers. Among 14 phenotypically confirmed ESBL isolates subjected to PCR, the *bla*_CTXM_ gene was identified in eight isolates (Table 5).

**Figure 2:**
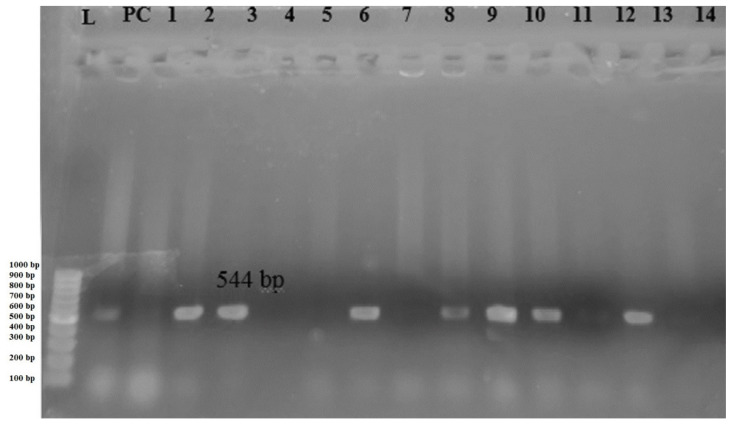
Gel electrophoresis of PCR amplified *bla*_CTX-M_ gene, Lane L: DNA ladder, Lane PC: Positive control, Lane 1–14: 544 bp PCR product of *bla*_CTX-M_ gene.

Gel electrophoresis of amplified blaCTX_-M_ gene is shown in Figure 2.

## 4. Discussion

An accurate and rapid diagnosis is important for the management and treatment of diseases. UTIs comprise a wide variety of clinical entities as a result of microbial invasion of tissues lining the urinary tract, extending from the renal cortex to the urethral meatus [28]. Resistance against antibiotics in clinically relevant bacteria is one of the most imminent threats to public health and especially to the most vulnerable patient populations.

In this current study, out of 217 mid-stream urine samples, 48 (22.12%) samples showed culture positivity. Among the total isolates, 46 (21.20%) were Gram negative bacteria. The incidence of Gram negative bacteria in the current study is markedly lower than the study conducted by other researchers, where >40% Gram negative bacteria had been recovered [29,30,31]. Among the total isolates, *E. coli* 37 (77.08%) was the most prevalent one. This result is in commensurate with some previous studies [4,32,33]. The higher prevalence of *E. coli* in UTIs might be due to the higher binding affinity to the glycoconjugate receptor of the uroepithelial cells [34].

The sensitivity and specificity of the urinary dipstick test were 43.75% and 77.51%, respectively, which is in tune with the findings of other investigators who have reported a sensitivity in between 28–48% [35,36]. A study conducted in Ethiopia has also reported a decrease in the sensitivity (42.9%) and increase in specificity (98.2%) of urine dipstick tests [11]. These values of sensitivity, specificity, positive predictive values and negative predictive values are comparable to our study. Positive nitrite tests suggest that nitrite has been produced from the reduction of nitrate by genera of the Enterobacteriaceae family [11]. When UTIs caused by microorganisms lack nitrite reductase, false negative results can occur [11].

Out of 48 isolates, 39 (25.00%) were from females and nine (14.75%) were from male patients. Similar results were observed by some researchers in Nepal, where 30–51.98% of positive isolates were from females and 18.83–48.01% of positive isolates were from males [37,38]. The results of this current study revealed that females were more susceptible to UTIs than males (*p* > 0.05). This finding is in alignment with some studies conducted by other researchers [39,40,41]. The increased incidence of UTIs in females may be due to favouring anatomic factors, hormonal changes and urodynamic disturbance occurring to age [42]. Even though the highest percentage of the UTI-suspected patients belonged to the age group 21–39, patients aged 60–79 were found to be more prone (29.17%) to develop UTIs (*p* < 0.05). This could be associated with a decreased immune system, which makes them susceptible to the infection. Moreover, out-patients were seen to be more likely (26.81%) to get UTI than in-patients (*p* < 0.05) in the present study. This is in line with the study done in the USA where the majority of isolates responsible for UTI were found in female out-patients [43].

This study showed amikacin (81.08%) to be the most effective drug against *E. coli* isolates. Similarly, nitrofurantoin (70.27%), gentamicin (62.16%) and piperacillin/tazobactam (62.16%) also showed good activity. This finding is similar to various studies [44,45]. The study demonstrated 62.16% of *E. coli* isolates resistant to cefazolin which is similar to the findings reported in a previous study [46]. A total of 33 (89.19%) *E. coli* isolates were MDR. The prevalence of MDR *E. coli* isolates in the current study was higher than a study done in Nepal [47] and in Bangladesh [48]. The haphazard use of antimicrobial drugs has resulted in the emergence and speedy dissemination of resistant bacterial strains. Encoding enzymes such as beta-lactamases and efflux pumps aid in conferring resistance to different antibiotics [49].

ESBL production among clinical *E. coli* isolates is an alarming public health issue these days. In the present study, 14 (37.84%) isolates were confirmed as ESBL isolates by phenotypic methods. This is quite lower than the study conducted in Southern Nepal, where 62.31% *E. coli* isolates were phenotypically confirmed as ESBL producers [38]. Such variation in the proportion of ESBL-producers in different parts of the country may be attributable to various factors such as socio-economic conditions and common practice of drug misuse. Furthermore, eight (21.62%) isolates carried the *bla*_CTX-M_ gene, which is lower than its prevalence reported in previous studies [47,50]. The distribution of ESBLs has evolved to a predominance of CTX-M enzymes, mainly with *E. coli* as one of the major carriers of ESBL encoding genes [51]. These higher rates of CTX-M among *E. coli* isolates may be associated with high mobilization of the encoding genes. Thus, the global dissemination of CTX-M producing strains highlights the need for their epidemiological monitoring and surveillance along with prudent use of antimicrobial agents.

## 5. Strengths and Limitations

Compared to the culture methods, the dipstick method (if it possesses the same sensitivity and specificity) can be useful in resource poor settings, basically of low-middle-income countries (LMICs) for rapid diagnosis and initiation of antibiotic therapy. Therefore, this study is one among the fewest scientific studies that attempted to analyse the potential use of dipstick test in place of routine culture methods for the diagnosis of UTIs. In addition, this study is another pivotal attempt to detect the *bla*_CTX-M_ genes that are responsible for conferring resistance to the Gram-negative uropathogens since there are further limited studies in molecular detection. The findings of this study may serve as an important tool for the clinicians, policy makers and researchers. Beside these strengths, this study also suffers from a number of limitations. This study involved a limited sample from a single tertiary care centre of Nepal. Therefore, the sample population and findings of the study may not be representative of larger population and geographical regions. In addition, due to constraints of logistic and molecular facilities, this study could not establish the antibiogram and resistant genotypes of uropathogens other than *E. coli*. Moreover, this study could not characterize the other members of ESBL genes of the same family (for instance, *bla*_SHV_) as well as the beta-lactamases of other Ambler classes. Furthermore, this study could not predict the origin and potential transmissibility of the detected genes. Therefore, future studies are recommended which can better address the limitations seen in our findings so that a clear picture can be drawn regarding the tools of diagnosis and burden of the AMR in the country.

## 6. Conclusions

The current study showed that Nitrite dipstick test results showed high specificity but low sensitivity besides being associated with several false positive and negative results. Hence, this test should not be considered as the diagnostic test for UTIs as far as culture methods are available. The study also revealed that *E.* *coli* was the most predominant bacteria causing urinary tract infections. More than one-third of the *E. coli* isolates were ESBL-producers, of which around half of the isolates harboured the *bla*_CTXM_ gene. Control of drug abuse, regular surveillance of ESBL-producers and implementation of hospital infection control policies to prevent the transmission of such isolates is definitely required.

## Figures and Tables

**Table 1:** Nucleotide sequence of the primer used in the detection of *bla*_CTX-M_ gene.

Gene	Primer (5′-3′)	Amplicon Size (bp)	Reference
CTX-M	F: 5′-TTTGCGATGTGCAGTACCAGTAA-3′	544	[26]
	R: 5′-CGATATCGTTGGTGGTGCCATA-3′		

**Table 2:** Sensitivity, specificity, positive predictive value and negative predictive value for urinary dipstick test.

Culture				
		Positive	Negative	Total
Dipstick test	Positive	21	38	59
	Negative	27	131	158
	Total	48	169	217

Sensitivity, specificity, positive predictive value and negative predictive value of urine dipstick test were found to be 43.75%, 77.51%, 35.59% and 82.91%, respectively.

**Table 3:** Association of UTIs with respect to various attributes.

Attributes		Sample Size	UTI Infection *n* (%)	*p*-Value
Gender	Male	61	9 (14.75)	0.103
	Female	156	39 (25.00)	
Age-group	1–19	37	9 (24.32)	0.019 *
	20–39	103	24 (23.30)	
	40–59	50	8 (16.00)	
	60–79	24	7 (29.17)	
	>80	3	0	
Department	In-patients	79	11 (13.92)	0.028 *
	Out-patients	138	37 (26.81)	

* Significant at 5% level of significance.

**Table 4:** Antibiotic susceptibility pattern of *E. coli*.

Antibiotics Used	Susceptibility Pattern		
	Sensitive *n* (%)	Intermediate *n* (%)	Resistant *n* (%)
Amikacin	30 (81.08)	0	7 (18.92)
Amoxyclav	17 (45.95)	1 (2.70)	19 (51.35)
Ciprofloxacin	16 (43.24)	1 (2.70)	20 (54.05)
Cotrimoxazole	17 (45.95)	0	20 (54.05)
Gentamicin	23 (62.16)	2 (5.40)	12 (32.43)
Cefixime	15 (40.54)	0	22 (59.46)
Nitrofurantoin	26 (70.27)	1 (2.70)	10 (27.03)
Cefazolin	13 (35.14)	1 (2.70)	23 (62.16)
Ceftriaxone	20 (54.05)	0	17 (45.95)
Cefotaxime	20 (54.05)	0	17 (45.95)
Ceftazidime	18 (48.65)	0	19 (51.35)
Nalidixic acid	13 (35.14)	2 (5.40)	22 (59.46)
Piperacillin/Tazobactam	23 (62.16)	0	14 (37.84)

**Table 5:** Prevalence of ESBL positive *E. coli* and *bla*_CTX-M_ gene.

Organism	Total Isolates	ESBL Producers		
		Presumptive *n* (%)	Confirmatory *n* (%)	*bla*CTX-M Gene *n* (%)
*E. coli*	37	19 (51.35)	14 (37.84)	8 (21.62)

## Data Availability

All the data pertaining to this study are within the manuscript.
